# GlcNAc6ST-1 regulates sulfation of *N*-glycans and myelination in the peripheral nervous system

**DOI:** 10.1038/srep42257

**Published:** 2017-02-10

**Authors:** Takeshi Yoshimura, Akiko Hayashi, Mai Handa-Narumi, Hirokazu Yagi, Nobuhiko Ohno, Takako Koike, Yoshihide Yamaguchi, Kenji Uchimura, Kenji Kadomatsu, Jan Sedzik, Kunio Kitamura, Koichi Kato, Bruce D. Trapp, Hiroko Baba, Kazuhiro Ikenaka

**Affiliations:** 1 Division of Neurobiology and Bioinformatics, National Institute for Physiological Sciences, National Institutes of Natural Sciences, Okazaki, Aichi 444-8787, Japan; 2 Department of Physiological Sciences, School of Life Sciences, SOKENDAI (The Graduate University for Advanced Studies), Hayama, Kanagawa 240-0193, Japan; 3 Department of Molecular Neurobiology, Tokyo University of Pharmacy and Life Sciences, Hachioji, Tokyo 192-0392, Japan; 4 Department of Structural Biology and Biomolecular Engineering, Graduate School of Pharmaceutical Sciences, Nagoya City University, Nagoya, Aichi 467-8603, Japan; 5 Department of Biochemistry, Graduate School of Medicine, Nagoya University, Nagoya, Aichi 466-8550, Japan; 6 Department of Chemical Engineering and Technology, Protein Crystallization Facility, Royal Institute of Technology, KTH, Stockholm 10044, Sweden; 7 Faculty of Health and Medical Care, Saitama Medical University, Hidaka, Saitama 350-1241, Japan; 8 Institute for Molecular Science and Okazaki Institute for Integrative Bioscience, National Institutes of Natural Sciences, Okazaki, Aichi 444-8787, Japan; 9 Department of Neurosciences, Lerner Research Institute, Cleveland Clinic, Cleveland, OH 44195, USA

## Abstract

Highly specialized glial cells wrap axons with a multilayered myelin membrane in vertebrates. Myelin serves essential roles in the functioning of the nervous system. Axonal degeneration is the major cause of permanent neurological disability in primary myelin diseases. Many glycoproteins have been identified in myelin, and a lack of one myelin glycoprotein results in abnormal myelin structures in many cases. However, the roles of glycans on myelin glycoproteins remain poorly understood. Here, we report that sulfated *N*-glycans are involved in peripheral nervous system (PNS) myelination. PNS myelin glycoproteins contain highly abundant sulfated *N*-glycans. Major sulfated *N*-glycans were identified in both porcine and mouse PNS myelin, demonstrating that the 6-*O*-sulfation of *N*-acetylglucosamine (GlcNAc-6-*O*-sulfation) is highly conserved in PNS myelin between these species. P_0_ protein, the most abundant glycoprotein in PNS myelin and mutations in which at the glycosylation site cause Charcot-Marie-Tooth neuropathy, has abundant GlcNAc-6-*O*-sulfated *N*-glycans. Mice deficient in *N*-acetylglucosamine-6-*O*-sulfotransferase-1 (GlcNAc6ST-1) failed to synthesize sulfated *N*-glycans and exhibited abnormal myelination and axonal degeneration in the PNS. Taken together, this study demonstrates that GlcNAc6ST-1 modulates PNS myelination and myelinated axonal survival through the GlcNAc-6-*O*-sulfation of *N*-glycans on glycoproteins. These findings may provide novel insights into the pathogenesis of peripheral neuropathy.

Rapid and efficient action potential propagation in vertebrates depends on axon ensheathment by a multilamellar membrane called myelin. Myelin, which is formed by Schwann cells in the peripheral nervous system (PNS) and by oligodendrocytes in the central nervous system (CNS), enwraps axons in segments that are separated by the nodes of Ranvier^1^,^2^,^3^. Recent studies have revealed that myelination tunes axonal functions, and vice versa^4^,^5^. Degeneration of demyelinated axons is a major cause of permanent neurological disability in primary human myelin diseases^6^. Glycoproteins are prominent components of plasma membranes, and as such, many glycoproteins have been identified in myelin where they play important roles in the formation, maintenance and degeneration of myelin sheaths^7^,^8^,^9^. However, the precise structures and roles of glycans on myelin glycoproteins remain largely unknown. Even though cell surface glycosylation is one of the key features universal to all eukaryotic cells, the degree of evolutionary conservation of glycans between taxa is very low in contrast to that of genetic and protein sequences^10^. Although the core structures of most types of glycans tend to be conserved across various taxa, within different mammalian species glycan structure is diverse^11^. However, interspecific comparative analyses of glycan structures have not been described in detail.

Circulating antibodies directed against specific glycoconjugates are associated with a variety of diseases, including Guillain-Barré syndrome, Fisher syndrome and chronic inflammatory demyelinating polyradiculoneuropathy^12^,^13^. Sulfated glycans are highly antigenic, acting as epitopes for autoantibodies in peripheral demyelinating neuropathy^7^,^14^,^15^. However, complete sulfated glycan structures as well as the function of myelin glycans remain largely unknown. Moreover, the enzyme responsible for myelin glycan sulfation remains to be identified.

In this study, we found that GlcNAc-6-*O*-sulfated *N*-glycans are highly conserved and abundant in PNS myelin among mammals. GlcNAc-6-*O*-sulfotransferase-1 (GlcNAc6ST-1) deficiency in mice resulted in a lack of sulfated *N*-glycans, and caused abnormal myelination and axonal degeneration in the PNS. These results suggest that the 6-*O*-sulfation of the GlcNAc residue by GlcNAc6ST-1 is critical for normal PNS myelination.

## Results

### Anionic *N*-glycans are highly abundant in PNS myelin compared to CNS myelin

**Figure 1:**
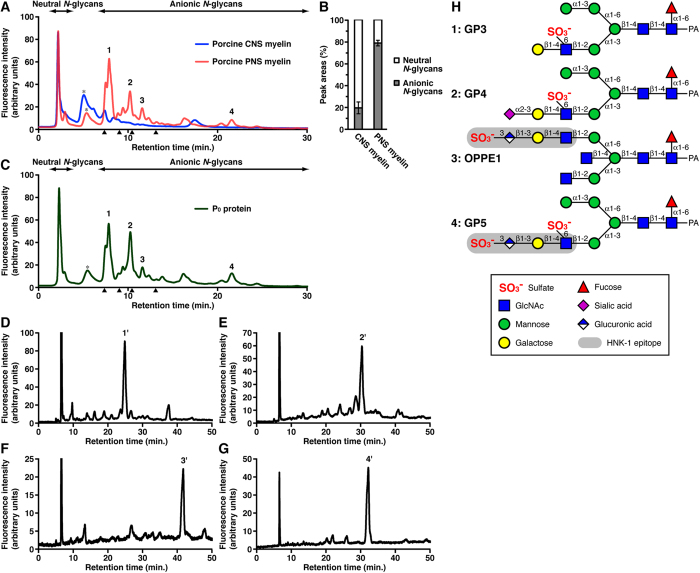
Sulfated *N*-glycans are abundant in porcine PNS and on P_0_ protein. (**A**) PA-*N*-glycans from porcine CNS (blue) and PNS (red) myelin were separated using a DEAE column. Peaks 1–4 were numbered in order of elution time for the anionic fractions. The asterisk indicates the peak derived from contaminants. Arrowheads indicate the elution positions of mono-, di-, tri-, and tetra-sialyl standard PA-oligosaccharides. (**B**) The percentages of neutral and anionic *N*-glycans from porcine CNS and PNS myelin were measured as the peak areas on DEAE HPLC; n = 4 for each group. Error bars indicate the mean ± SD. (**C**) PA-*N*-glycans from purified porcine P_0_ protein were separated using a DEAE column. The elution positions of peaks 1–4 coincided with those from porcine PNS myelin. The peak 1–4 fractions were collected individually. (**D**–**G**) The anionic fractions of peaks 1 (**D**), 2 (**E**), 3 (**F**) and 4 (**G**) from P_0_ protein were individually analyzed by RP-HPLC. Main peaks 1′ (**D**), 2′ (**E**), 3′ (**F**) and 4′ (**G**) were numbered. (**H**) The *N*-glycans of peaks 1′–4′ in Fig. 1D–G were identified as sulfated *N*-glycans. All structures are shown as pyridylaminated (PA-) forms.

We developed a systematic method to purify and analyze *N*-glycans from small tissue samples^16^,^17^. Moreover, our method is highly effective at analyzing water-insoluble samples including myelin proteins. To determine the *N*-glycan profiles in PNS and CNS myelin, both types of myelin were purified from porcine peripheral spinal roots and spinal cords, respectively. Purified *N*-glycans from PNS and CNS myelin were tagged with the fluorophore, 2-aminopyridine^16^. The pyridylaminated (PA)-*N*-glycans were then analyzed by high performance liquid chromatography (HPLC) using an anion exchange DEAE column, which separates sugar chains based on their negative charge (Fig. 1A). Neutral *N*-glycans are first eluted in the non-adsorbed fraction, and then anionic *N*-glycans are eluted in subsequent fractions. The amount of *N*-glycan was measured as the peak area in the HPLC chromatograms. As compared with porcine CNS myelin, porcine PNS myelin contained abundant anionic *N*-glycans harbored on glycoproteins (Fig. 1B).

To examine which glycoproteins harbor anionic *N*-glycans in PNS myelin, we focused on P_0_ protein, as glycoprotein P_0_ is the most abundant PNS protein accounting for 20% to over 50% of the total protein within PNS myelin^18^,^19^. P_0_ protein was purified from porcine PNS myelin by immobilized metal ion adsorption chromatography^20^, from which *N*-glycans were released and purified. PA-*N*-glycans from P_0_ protein were analyzed by DEAE HPLC (Fig. 1C). The *N*-glycan elution profile from P_0_ protein was similar to that from PNS myelin, and the retention times for major peaks 1–4 from P_0_ protein were identical to those from PNS myelin.

Major *N*-glycans from bovine PNS myelin have been reported^21^,^22^,^23^,^24^,^25^, and the *N*-glycan elution profile from bovine PNS myelin by DEAE HPLC was similar to that from porcine PNS myelin (Supplementary Fig. S1A). To identify porcine peak 1–4 *N*-glycans, the anionic fractions of peaks 1–4 from P_0_ protein were individually collected and further analyzed by reverse-phase (RP) HPLC (Fig. 1D–G). The four major *N*-glycans in bovine PNS myelin, GP3-5 and OPPE1, were used as standard sugar chains, and the *N*-glycans of main peaks 1′, 2′, 3′, and 4′ were identified as sulfated *N*-glycans, GP3, GP4, OPPE1, and GP5 (Fig. 1H). Thus, both porcine and bovine PNS myelin have abundant anionic *N*-glycans on their glycoproteins, especially sulfated *N*-glycans, compared to CNS myelin. In porcine PNS myelin, *N*-glycans on the most abundant protein P_0_ are highly sulfated.

### GlcNAc-6-*O*-sulfation of *N*-glycans in PNS myelin is highly conserved across mammalian species

**Figure 2:**
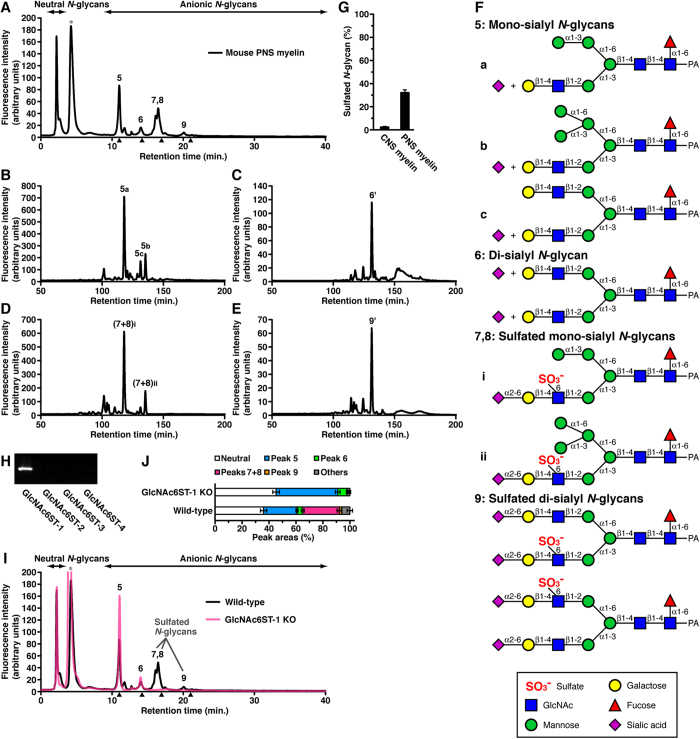
GlcNAc6ST-1 exclusively catalyzes sulfation of *N*-glycans in mouse PNS myelin. (**A**) PA-*N*-glycans from young adult mouse PNS myelin were analyzed by DEAE HPLC. Peaks 5–9 were numbered in order of elution time from the anionic fractions. The elution positions of peaks 5 and 6 were mono- and di-sialyl *N*-glycans, respectively. The peak 5–9 fractions were collected and further analyzed individually. The asterisk indicates the peak derived from contaminants. Arrowheads indicate the elution positions of mono-, di-, tri-, and tetra-sialyl standard PA-oligosaccharides. (**B**–**E**) The anionic fractions of peaks 5 (**B**), 6 (**C**), 7 + 8 (**D**) and 9 (**E**) from mouse PNS myelin were individually analyzed by NP-HPLC after desialylation (peaks 5 and 6) and desulfation (peaks 7 + 8 and 9). The major peaks 5a, 5b, 5c (**B**), 6′ (**C**), (7 + 8)i, (7 + 8)ii (**D**) and 9′ (**E**) were numbered. (**F**) The *N*-glycans of peaks 5a, 5b, 5c (**B**), 6′ (**C**), (7 + 8)i, (7 + 8)ii (**D**) and 9′ (**E**) in Fig. 2B–E were further analyzed (Supplementary Fig. S3), and the *N*-glycan structures from peaks 5–6 were identified. (**G**) The percentages of sulfated *N*-glycans from mouse CNS and PNS myelin were measured. Error bars indicate the mean ± SD (CNS myelin, n = 4; PNS myelin, n = 7). (**H**) The mRNA expression levels of four GlcNAc6STs in sciatic nerves were analyzed by RT-PCR. (**I**) PA-*N*-glycans from PNS myelin of young adult GlcNAc6ST-1-KO mice (red) were separated using a DEAE column. The elution positions of peaks 5–8 coincided with those from PNS myelin of WT mice (black; Fig. 2A). (**J**) The percentages of *N*-glycans from PNS myelin of young adult WT and GlcNAc6ST-1-KO mice were measured as the peak areas on DEAE HPLC. Error bars indicate the mean ± SD (WT mice, n = 7; GlcNAc6ST-1-KO mice, n = 3).

We next examined *N*-glycan profiles in mouse PNS and CNS myelin by DEAE HPLC (Fig. 2A and Supplementary Fig. S1B). Mouse PNS myelin had abundant anionic *N*-glycans, similar to porcine PNS myelin, while little anionic *N*-glycans were detected in mouse CNS myelin. The *N*-glycan elution profile from rat sciatic nerves was similar to that from mouse PNS myelin (Supplementary Fig. S1C). It is noteworthy that the anionic *N*-glycan elution profile from mouse PNS myelin was less similar to that from porcine PNS myelin (Figs 1A and 2A). To identify major peak 5–9 *N*-glycans from mouse PNS myelin, the anionic fractions of these peaks were individually collected and further analyzed. After desialylation (peaks 5 and 6 in Fig. 2A, Supplementary Fig. S2A and S2B) and desulfation (peaks 7 + 8 and 9 in Fig. 2A, Supplementary Fig. S2C and S2D), the *N*-glycans from peaks 5–9 became neutral and were analyzed by normal-phase (NP) HPLC (Fig. 2B–E). The *N*-glycans of the major peaks were further analyzed and identified by RP-HPLC, and their elution times were compared with those of known standards (Supplementary Fig. S3). The *N*-glycan structures from peaks 5–6 are shown in Fig. 2F (for details see Methods). The amount of sulfated *N*-glycans was calculated. As compared with mouse CNS myelin, mouse PNS myelin contained highly abundant sulfated *N*-glycans on glycoproteins (Fig. 2G).

The structure of the main *N*-glycans in the neutral fraction from mouse PNS myelin in Fig. 2A was determined, and is shown in Supplementary Fig. S4 (for details see Methods).

### GlcNAc6ST-1 sulfates *N*-glycans in mouse PNS myelin

Even though the precise sulfated *N*-glycan structures in mouse PNS myelin are not the same as those in porcine PNS myelin (e.g., there is a different sialyl linkage between GP4 in Fig. 1H and i in Fig. 2F), the 6-*O*-sulfation at the GlcNAc residue on the *N*-glycans is highly conserved between pig and mouse PNS myelin. The sulfation modification at the C6 position of the GlcNAc residue (GlcNAc-6-*O*-sulfation) is mediated by GlcNAc6STs, and four members of the GlcNAc6ST family have been cloned in mice^26^,^27^,^28^. We examined the transcript expression of GlcNAc6STs by reverse transcription (RT)-PCR in 12-week-old mouse PNS myelin (Fig. 2H). GlcNAc6ST-1 mRNA was detected in mouse sciatic nerves, whereas mRNAs encoding GlcNAc6ST-2, 3 and 4 were not detected in our experimental conditions. Additionally, GlcNAc6ST-1 mRNA was detected in the mouse CNS at a comparable amount to that in the PNS (Supplementary Fig. S5).

To examine whether GlcNAc6ST-1 catalyzes 6-*O*-sulfation of GlcNAc on *N*-glycans in PNS myelin, *N*-glycans from GlcNAc6ST-1-knockout (KO) mouse PNS myelin were analyzed by DEAE HPLC. Sulfated *N*-glycans (peaks 7–9) from PNS myelin of young adult (Fig. 2I) and adult (Supplementary Fig. S6) GlcNAc6ST-1-null mice were not detected, whereas the levels of non-sulfated sialyl *N*-glycans (peaks 5 and 6) were increased compared to wild-type (WT) (Fig. 2J). *N*-glycans of peaks 5 and 6 were desulfated forms of peaks 7–9 (Fig. 2F). These results indicate that GlcNAc6ST-1 sulfates *N*-glycans, and there is no compensation mechanism for the GlcNAc-6-*O*-sulfation of *N*-glycans in mouse PNS myelin.

### *N*-glycans on P_0_ protein are not sulfated in the CNS

Proteolipid protein (PLP) is an abundant protein found in CNS myelin, and replaced P_0_ as the major CNS myelin structural protein during CNS myelin evolution^29^,^30^. The myelin compaction phenotype in PLP-null mice was rescued by transgene expression of P_0_ protein (P_0_-CNS mice), but the axonal degeneration phenotype was exaggerated in P_0_-CNS mice^31^. To determine the glycosylation profile of P_0_ protein in CNS myelin, we analyzed *N*-glycans in CNS myelin from P_0_-CNS mice (Supplementary Fig. S7). Since P_0_ was expressed as a major protein in the CNS myelin of P_0_-CNS mice^31^, the *N*-glycan expression profile would be affected if the *N*-glycans on P_0_ proteins were sulfated as in PNS myelin. The DEAE HPLC elution profile of *N*-glycans in CNS myelin from P_0_-CNS mouse brains was similar to that from WT and PLP-null mouse brains. The *N*-glycans forming the major peaks in the anionic fraction from mouse brains were sialylated but not sulfated as described previously^17^. These results suggest that *N*-glycans on P_0_ protein are not sulfated when P_0_ is expressed in CNS myelin.

### GlcNAc6ST-1-KO mice display abnormal myelination and axonal degeneration in the PNS

**Figure 3:**
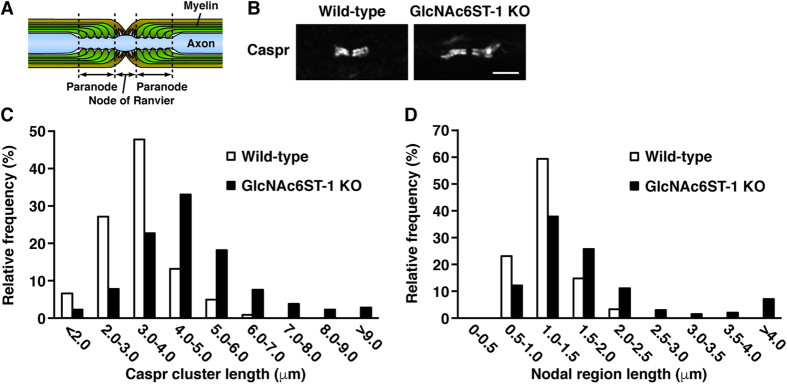
GlcNAc6ST-1 deficiency in mice causes abnormal myelination in the PNS. (**A**) The schematic drawing represents structural and functional domains in a longitudinal section of a myelinated axon around the node of Ranvier. (**B**) Longitudinal sections of sciatic nerves from young adult WT and GlcNAc6ST-1-KO mice were immunostained using antibodies against Caspr, a paranodal marker. Scale bar, 5 μm. (**C**) The Caspr-stained paranodal lengths of sciatic nerves from young adult WT (n = 122) and GlcNAc6ST-1-KO (n = 398) mice were measured. (**D**) The nodal gap lengths between paranodal Caspr clusters were measured in sciatic nerves from young adult WT (n = 61) and GlcNAc6ST-1-KO (n = 199) mice.

Peripheral nerves of P_0_-KO mice are characterized by severe hypomyelination and axonal degeneration^32^,^33^. Myelinated axons are segregated into distinct domains that include the nodes of Ranvier and their flanking paranodal regions^2^,^3^ (Fig. 3A). To investigate the role of sulfated *N*-glycans in the PNS, longitudinal sections of sciatic nerves from WT and GlcNAc6ST-1-KO mice were immunostained using antibodies against Caspr as a paranodal marker^34^ (Fig. 3B). The lengths of the paranodal regions and the nodal gaps between paranodes were elongated in GlcNAc6ST-1-KO mice compared to WT mice (Fig. 3C and D). Thus, GlcNAc6ST-1 deficiency in mice causes abnormal myelin structures. These results indicate that GlcNAc6ST-1 modulates myelination in the PNS.

**Figure 4:**
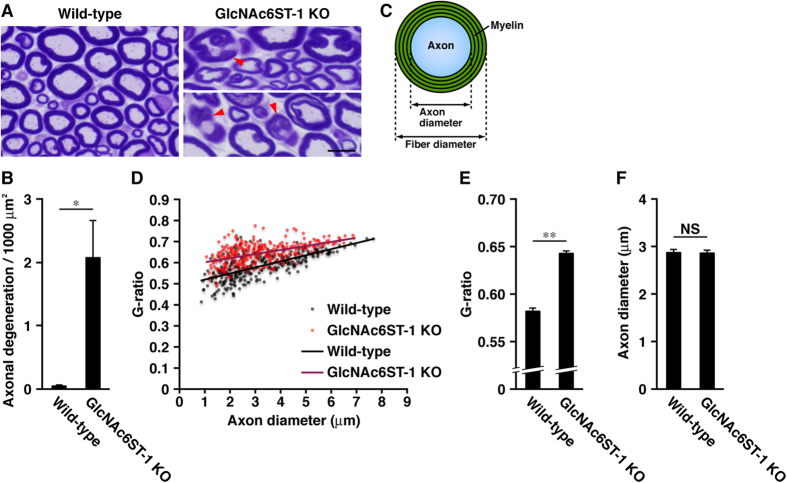
GlcNAc6ST-1-knockout mice display axonal degeneration in the PNS. (**A**) Semi-thin cross sections of sciatic nerves from young adult WT and GlcNAc6ST-1-KO mice were stained with toluidine blue. Arrowheads indicate degenerating axons. Scale bar, 10 μm. (**B**) The numbers of degenerating axons were counted in the sciatic nerves of young adult WT and GlcNAc6ST-1-KO mice. Unpaired, two-tailed Student’s t-test; *P < 0.05. Data are shown as the mean ± SEM (n = 4 mice for each group; areas greater than 320,000 μm^2^ were counted). (**C**) G-ratio calculation for a myelinated axon. G-ratio = axon diameter/fiber diameter. (**D**) Scatter plot displays g-ratios as a function of axon diameter measured in the sciatic nerves of young adult WT (black) and GlcNAc6ST-1-KO (red) mice. (**E**) Myelin sheath thickness was quantified using the g-ratio in sciatic nerves of WT and GlcNAc6ST-1-KO mice. (**F**) Average diameters of myelinated axons in the sciatic nerves of young adult WT and GlcNAc6ST-1-KO mice. Unpaired, two-tailed Student’s t-test; **P < 0.01. Error bars indicate the mean ± SEM (>300 myelinated fibers from each group; n = 3 mice from each group). NS, not significant.

We next stained transverse semi-thin sections of sciatic nerves from 18-week-old young adult WT and GlcNAc6ST-1-null mice with toluidine blue (Fig. 4A). GlcNAc6ST-1-null mice displayed axonal degeneration in the sciatic nerves (Fig. 4A, arrowheads, and Fig. 4B). We also measured the g-ratios (axon diameters/fiber diameters) of myelinated axons (Fig. 4C–F), and found that the average g-ratio in young adult GlcNAc6ST-1-null mice was significantly increased (Fig. 4E). These results indicate that GlcNAc6ST-1 deficiency in young adult mice causes abnormal myelination and axonal degeneration.

### Ultrastructure of GlcNAc6ST-1-KO sciatic nerves

**Figure 5:**
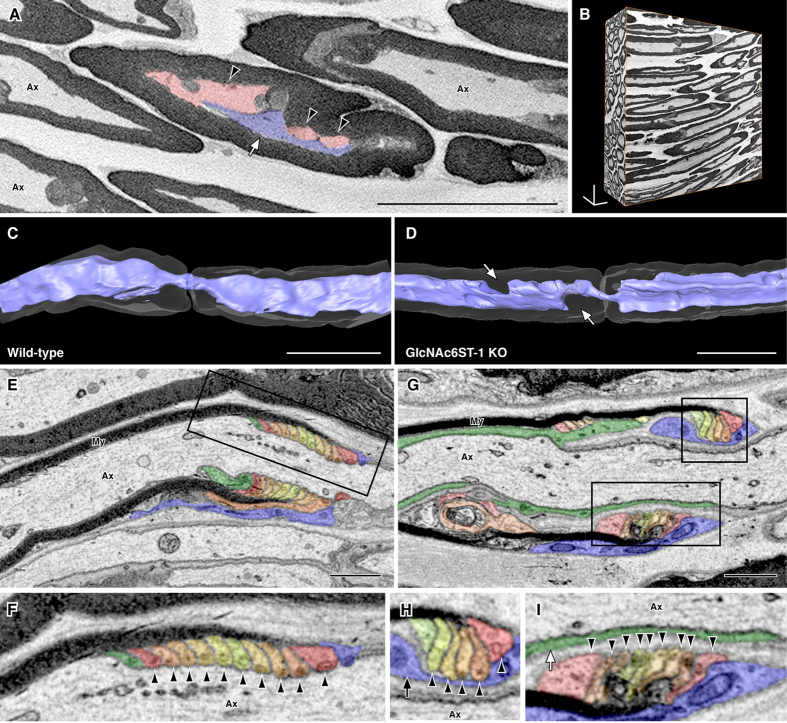
Abnormal axonal morphology and disorganized paranodes of myelinated axons in GlcNAc6ST-1-KO mice revealed by serial image acquisition with SBF-SEM. (**A**–**D**) One of serial images of GlcNAc6ST-1-KO (**A**) and 3D reconstructions of WT (**C**) and GlcNAc6ST-1-KO (**B**,**D**) sciatic nerves. There were vacuolar structures (red, arrowheads) between the myelin sheath and axolemma in the myelinated axons (blue, white arrow) of GlcNAc6ST-1-KO mice (**A**). 3D reconstructions show abnormal thinning of axoplasm in the axons of GlcNAc6ST-1-KO mice (arrows, **D**). Ax, axon. Scale bars: 20 μm. (**E**–**I**) In the normal myelinated axons (**E**,**F**), paranodal loops (arrowheads, variously colored, **F**) were tightly attached to the axolemma. In the myelinated axons of GlcNAc6ST-1-KO mice (**G**–**I**), paranodal loops were detached from the axolemma. The outer collar (arrow, blue, **H**) and inner cytoplasmic tongue (white arrow, green, **I**) of the myelinating Schwann cell were located between the paranodal loops and the axon. My: compact myelin. Areas indicated with rectangles (**E**,**G**) are magnified (**F**,**H**,**I**). Scale bars: 1 μm.

The abnormal morphology in sciatic nerves of GlcNAc6ST-1-KO mice was further investigated using serial block face-scanning electron microscopy (SBF-SEM) to acquire serial electron microscopic images. Among normal appearing axons, some myelinated axons of adult GlcNAc6ST-1-null mice had vacuolar structures between the myelin sheath and axolemma (Fig. 5A), and showed abnormal thinning of the axoplasm, whereas abnormal structures were not observed in those of WT mice (Fig. 5B–D). While paranodal loops were tightly attached to the axolemma in the normal myelinated axons (Fig. 5E and F), some myelinated axons of young adult GlcNAc6ST-1-KO mice showed that paranodal loops were detached from the axolemma, and outer collar and inner cytoplasmic tongue of the myelinating Schwann cell were located between the paranodal loops and the axon (Fig. 5G–I). These results indicate that GlcNAc6ST-1 deficiency in mice leads to abnormal morphology in the PNS.

## Discussion

**Figure 6:**
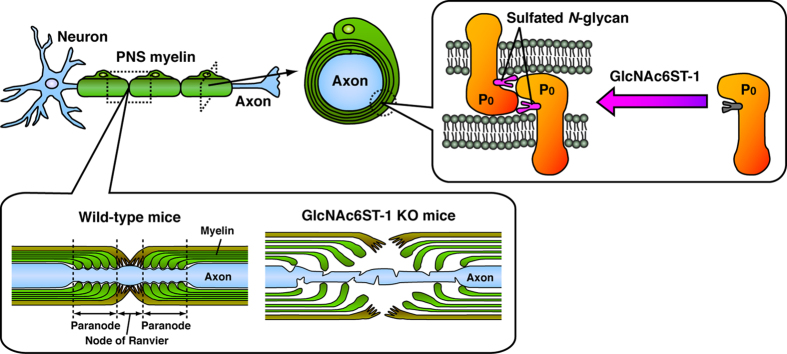
Schematic representation illustrating regulation of PNS myelination by GlcNAc6ST-1. GlcNAc6ST-1 sulfates *N*-glycans on P_0_ protein. GlcNAc6ST-1-KO mice fail to synthesize sulfated *N*-glycans and exhibit abnormal myelination and axonal degeneration in the PNS. Sulfated *N*-glycans on P_0_ protein may contribute to the stabilization of compact PNS myelin via homophilic adhesion.

In the present study we showed that GlcNAc6ST-1 almost exclusively catalyzes sulfation of *N*-glycans on glycoproteins in mouse PNS myelin (Fig. 2). We further found that GlcNAc6ST-1 modulates myelination and myelinated axonal survival in the mouse PNS (Figs 3,4,5 and 6). Surprisingly, GlcNAc-6-*O*-sulfated *N*-glycans are abundant and well conserved in mammalian PNS myelin (Figs 1 and 2 and Supplementary Fig. S1), whereas levels of sulfated *N*-glycans are generally very low^35^, as described for CNS myelin. Therefore, sulfated *N*-glycans play important roles in the PNS.

A unique sulfated glycan structure, human natural killer-1 (HNK-1), is a sulfated glucuronic acid attached to the non-reducing terminal of an *N*-acetyllactosamine residue, and one of the epitopes for autoantibodies in peripheral demyelinating neuropathy^7^,^14^,^15^. The HNK epitope is reported to be present in variable amounts, with little or no presence in rodents, and much higher levels in bovine and human PNS myelin^36^,^37^,^38^,^39^,^40^. Our results on the amount of HNK-1-containing glycans (Figs 1 and 2 and Supplementary Fig. S1) are consistent with these previous reports. However, we also demonstrated that the GlcNAc-6-*O*-sulfation of *N*-glycans on glycoproteins was highly conserved in PNS myelin between rodents and other mammals. Therefore, our results suggest that GlcNAc-6-*O*-sulfated *N*-glycans share a common key role in PNS myelination among mammals, and HNK-1-containing glycans may perform a specific function in human, porcine and bovine PNS myelin.

Defects in Schwann cell genes can cause dysmyelinating peripheral neuropathies, which can include axonal degeneration^6^. Here we showed that loss of sulfated *N*-glycans by GlcNAc6ST-1 deficiency results in abnormal myelination and axonal degeneration in the PNS (Figs 3,4,5 and 6). P_0_ protein is a Schwann cell-specific glycoprotein with a single *N*-glycosylation site, and is the most abundant PNS myelin protein^41^. P_0_ protein contributes to the formation and maintenance of myelin compaction by homophilic interactions, and the glycosylation of P_0_ protein is essential for homophilic adhesion^42^,^43^,^44^. A point mutation in the glycosylation site of P_0_ protein abolishes P_0_ glycosylation^44^. However, GlcNAc6ST-1 deletion inhibited only GlcNAc-6-*O*-sulfation, whereas the *N*-glycan backbone structure was maintained (Fig. 2F,I and J). P_0_ protein carrying high-mannose type sugar residues has been shown to be non-adhesive^43^. We demonstrated that the sulfated *N*-glycan on P_0_ protein is a hybrid or complex type glycan, not a high-mannose type glycan (Fig. 1C and H). We propose that sulfated *N*-glycans on P_0_ proteins stabilize compact PNS myelin via homophilic adhesion (Fig. 6), though we cannot exclude the possibility that other myelin proteins with sulfated *N*-glycans are involved in myelination.

Charcot-Marie-Tooth (CMT) disease is the most common inherited disorder of the PNS, and CMT type 1B is caused by over 200 mutations in the gene encoding P_0_ protein^45^. Clinically, different P_0_ protein mutations cause different forms of CMT: dominantly inherited demyelinating, dominant axonal, or dominant intermediate forms^41^,^46^. Patients with P_0_ protein mutations near the glycosylation site develop an axonal CMT (CMT2), and patients with P_0_ protein mutations at the glycosylation site develop a demyelinating CMT (CMT1B)^41^. P_0_-null mice display severe hypomyelination and axonal degeneration in the PNS^32^,^33^. Mouse PNS demyelination induced by intraneural injection of lysolecithin showed elongated Caspr immunostaining^47^. Sural nerve biopsies from patients with hereditary neuropathies had similar elongated Caspr immunostaining^48^,^49^. We also found that PNS nerves from GlcNAc6ST-1-null mice displayed elongated Caspr immunostaining and axonal degeneration (Figs 3 and 4). These results suggest that depletion of GlcNAc6ST-1 reduces PNS myelin compaction and alters paranodal structures (Fig. 6). Subsequently, the lack of Schwann cell support causes axonal degeneration. Therefore, GlcNAc6ST-1 may be involved in the pathogenesis of peripheral neuropathy.

With the appearance of reptiles/aves, the function of PLP became fully established, allowing the silent dropout of P_0_ from CNS myelin^29^,^30^. In fish, P_0_ protein mediates both CNS and PNS myelin compaction^29^,^30^. Moreover, *N*-glycans on fish P_0_ in both CNS and PNS myelin are sulfated^50^. It would be interesting to identify and compare *N*-glycans on fish P_0_ in the CNS and PNS from an evolutionary point of view. When PLP was experimentally replaced by P_0_ in mouse CNS myelin, this PLP-P_0_ shift resulted in degeneration of myelinated axons, severe neurological disability, and reduced myelin internode length, whereas PLP/P_0_-CNS mice containing PLP and P_0_ do not seem to develop these phenotypes and pathologies^31^. Although GlcNAc6ST-1 is present and active in producing keratan sulfate in the mouse CNS^26^,^51^,^52^ (Supplementary Fig. S5), P_0_ protein in CNS myelin does not seem to harbor sulfated *N*-glycans in P_0_-CNS mice (Supplementary Fig. S7). These results thus suggest that GlcNAc6ST-1 is involved in the synthesis of keratan sulfate in the CNS, but not toward *N*-glycan sulfation. It is possible that there are different mechanisms by which GlcNAc6ST-1 recognizes substrates between the mouse CNS and PNS. Further studies are necessary to address this issue.

Taken together, our results support a model where GlcNAc6ST-1 regulates PNS myelination through sulfation of *N*-glycans on P_0_ protein. Our findings may provide novel insights into the pathogenesis of peripheral neuropathy.

## Methods

Detailed procedures and reagent information are presented in the Supplementary Information.

### Animals

All experimental procedures were approved by the Animal Care and Use Committee of National Institute for Physiological Sciences and Nagoya University Graduate School of Medicine, and conducted in accordance with the Guidelines and Regulations for the Care and Use of Experimental Animals by National Institute for Physiological Sciences and Nagoya University Graduate School of Medicine. GlcNAc6ST-1-KO, P_0_-CNS and PLP-null mice were generated as described previously^31^,^53^. Wistar rats and 10–18-week-old young adult and 30–33-week-old adult ICR mice were purchased from Japan SLC (Hamamatsu, Japan).

### Purification of myelin fractions and P_0_ protein

Purification of myelin fractions and P_0_ protein was performed as described previously^20^,^54^,^55^,^56^. Additional details are provided in the Supplementary Information.

### *N*-glycan purification, pyridylamination, and analysis for identification

*N*-glycan purification and pyridylamination were performed as described previously^16^,^17^. PA-*N*-glycans were analyzed by HPLC using the following columns: DEAE (TSKgel DEAE-5PW, Tosoh, Tokyo, Japan), NP (Shodex Asahipak NH2P-50 4E, Showa Denko, Tokyo, Japan) and RP (CAPCELL PAK C18 SG120, Shiseido, Tokyo, Japan or Develosil C30-UG-5, Nomura Chemical, Seto, Japan). Additional details are provided in the Supplementary Information.

### Immunofluorescence studies

Immunostaining was performed as described previously^57^. Additional details are provided in the Supplementary Information.

### Morphological studies

After the sciatic nerves were fixed, dehydrated and embedded, cross-sections of 0.7 μm thickness were cut and stained with 0.5% toluidine blue. Sections were observed using a light microscope. Additional details are provided in the Supplementary Information.

### SBF-SEM imaging and analyses

The imaging and 3D ultrastructural analyses were performed as described previously^58^,^59^. Additional details are provided in the Supplementary Information.

### Statistics

Statistical significance was determined by unpaired, two-tailed Student’s t-tests. P-values of <0.05 were considered statistically significant. Data were collected and processed randomly, and were analyzed using Microsoft Excel.

## Additional Information

**How to cite this article:** Yoshimura, T. *et al*. GlcNAc6ST-1 regulates sulfation of *N*-glycans and myelination in the peripheral nervous system. *Sci. Rep.* **7**, 42257; doi: 10.1038/srep42257 (2017).

**Publisher's note:** Springer Nature remains neutral with regard to jurisdictional claims in published maps and institutional affiliations.

## Supplementary Material

Supplementary Information
