# Supplementary material for: GlcNAc6ST-1 regulates sulfation of N-glycans and myelination in the peripheral nervous system
Source: Sci Rep. 2017 Feb 10;7:42257. doi: 10.1038/srep42257 (PMC5301494; doi:10.1038/srep42257)

Supplementary Information

GlcNAc6ST-1 regulates sulfation of *N*-glycans and myelination in the peripheral nervous system

Takeshi Yoshimura[1, 2, †, *], Akiko Hayashi[3, *], Mai Handa-Narumi[1, 2], Hirokazu Yagi[4], Nobuhiko Ohno[1, 2], Takako Koike[1], Yoshihide Yamaguchi[3], Kenji Uchimura[5], Kenji Kadomatsu[5], Jan Sedzik[1, 6, 10], Kunio Kitamura[7], Koichi Kato[4, 8], Bruce D. Trapp[9], Hiroko Baba[3] & Kazuhiro Ikenaka[1, 2, *]

[1]Division of Neurobiology and Bioinformatics, National Institute for Physiological Sciences, National Institutes of Natural Sciences, Okazaki, Aichi 444-8787, Japan. [2]Department of Physiological Sciences, School of Life Sciences, SOKENDAI (The Graduate University for Advanced Studies), Hayama, Kanagawa 240-0193, Japan. [3]Department of Molecular Neurobiology, Tokyo University of Pharmacy and Life Sciences, Hachioji, Tokyo 192-0392, Japan. [4]Department of Structural Biology and Biomolecular Engineering, Graduate School of Pharmaceutical Sciences, Nagoya City University, Nagoya, Aichi 467-8603, Japan. [5]Department of Biochemistry, Graduate School of Medicine, Nagoya University, Nagoya, Aichi 466-8550, Japan. [6]Department of Chemical Engineering and Technology, Protein Crystallization Facility, Royal Institute of Technology, KTH, Stockholm 10044, Sweden. [7]Faculty of Health and Medical Care, Saitama Medical University, Hidaka, Saitama 350-1241, Japan. [8]Institute for Molecular Science and Okazaki Institute for Integrative Bioscience, National Institutes of Natural Sciences, Okazaki, Aichi 444-8787, Japan. [9]Department of Neurosciences, Lerner Research Institute, Cleveland Clinic, Cleveland, OH 44195, USA. [10]Deceased May 18, 2015. [†]Present address: Department of Child Development and Molecular Brain Science, United Graduate School of Child Development, Osaka University, 2-2 Yamadaoka, Suita, Osaka, 565-0871, Japan. [*]These authors contributed equally to this work. Correspondence and requests for materials should be addressed to T.Y. (email: tyoshimu@ugscd.osaka-u.ac.jp) or K.I. (email: ikenaka@nips.ac.jp)

Supplementary Methods

Materials and chemicals

Anhydrous hydrazine was purchased from Tokyo Chemical Industry (Tokyo, Japan), 2-aminopyridine was from Kanto Chemical (Tokyo, Japan), and dimethylamine borane was from Wako (Osaka, Japan). Graphite carbon columns (GL-Pak carbograph, Cat. No. 5010–23005) were purchased from GL Science (Tokyo, Japan). Cellulose cartridge columns were from Takara Bio (Cat. No. 4404; Otsu, Japan) and GL Science (Cat. No. 5010–11130). PA-sugar chains used as a standard were purchased from Takara Bio and Seikagaku Corporation (Tokyo, Japan). Monosialo, disialo, trisialo and tetrasialo PA-oligosaccharides were from Takara Bio (Cat. No. 4122, 4123, 4124 and 4125).

Purification of myelin fractions and P_0 protein

Purification of myelin fractions and P_0 protein was performed as described previously[1-4]. Porcine PNS and CNS myelin were purified from adult porcine peripheral spinal roots and spinal cords, respectively. Porcine spinal roots and spinal cords were obtained from the local slaughterhouse (JA Aichi, Toyota, Japan). Mouse PNS and CNS myelin were purified from mouse sciatic nerves and brains, respectively. Purified porcine PNS myelin was used for purification of P_0 protein.

Purification and pyridylamination of sugar chains

N-glycan purification and pyridylamination were performed as described previously[5,6]. Briefly, after acetone precipitation, samples were lyophilized before use. Each lyophilized sample (2 mg) was hydrazinolyzed (100ºC, 10 hrs). *N*-glycan purification and in-column *N*-acetylation was performed using graphite carbon columns. The reducing ends of the liberated glycans were tagged with the fluorophore 2-aminopyridine to aid detection via HPLC analyses. Excess reagents were removed and PA-*N*-glycans were purified using cellulose columns.

Desialylation and desulfation of *N*-glycans

Sialylated PA-*N*-glycans were treated with neuraminidase (Nacalai Tesque, Kyoto, Japan) at 37ºC for 14 hrs in 50 mM ammonium acetate (pH 5.0) to cleave sialic acids, followed by heating at 100ºC for 5 min and

filtering through a 0.20 μm spin filter (Ultrafree-MC LG, Millipore, Billerica, MA). α2,3-sialidase (New England BioLabs, Ipswich, MA), specific for α2,3 linked *N*-acetylneuraminic acid residues, was incubated with PA-*N*-glycans for 14 hrs at 37°C in 50 mM sodium citrate (pH 6.0), 100 mM NaCl, and 100 μg/ml bovine serum albumin (BSA). After heating at 100°C for 5 min, the reaction mixture was centrifuged at 2,300 x g for 10 min, followed by filtering through a 0.20 μm spin filter. Desulfation for dried samples was performed in 50 mM anhydrous methanol/HCl at 37ºC for 2 hrs. After mild methanolysis, the samples were dried under vacuum.

_N_-glycan analysis and separation by HPLC

Analyses of PA-*N*-glycans using HPLC were performed as described previously[5,6]. To separate neutral and anionic *N*-glycans, PA-*N*-glycans were passed through an anion-exchange DEAE column (TSKgel DEAE-5PW, Tosoh, Tokyo, Japan) using HPLC. Anion-exchange column HPLC purification was performed at a flow rate of 1.0 ml/min at room temperature. The mobile phase consisted of solvent A (distilled water adjusted to pH 9.0 with aqueous ammonia) and solvent B (0.5 M ammonium acetate titrated to pH 9.0 with aqueous ammonia). The column was equilibrated with solvent A. After a sample had been injected, solvent B was 0% in the first 5 min, and then increased linearly to 60% in the next 37 min. For analysis of porcine samples, P_0-CNS and PLP-null mouse samples, solvent B was 0% in the first 2 min, and then increased linearly to 12% for 3 min and 100% in the next 44 min. PA-sugar chains were detected at excitation and emission wavelengths of 310 nm and 380 nm, respectively (FP-2025 Plus, Jasco Corporation, Hachioji, Japan). Peaks between neutral and anionic *N*-glycans are derived from contaminants that are not removed during *N*-glycan purification[6].

RP-HPLC for anionic PA-*N*-glycans was performed on a CAPCELL PAK C18 column (SG120, Cat. No. 12512; Shiseido, Tokyo, Japan) at a flow rate of 0.6 ml/min at 45ºC. Solvent C consisted of 0.6% acetic acid buffer (pH 4.0) containing 0.28% triethylamine, and solvent D consisted of solvent C containing 5% acetonitrile. The column was equilibrated with mixtures of solvent C and solvent D (initial ratio 95:5) that were increased linearly to 100% in 60 min. After that, the ratio of 100% of solvent D was kept for 1 min. PA-sugar chains were detected at an excitation wavelength of 310 nm and an emission wavelength of 380 nm.

Neutral PA-*N*-glycans of varying sizes were separated by HPLC using a NP-column (Shodex Asahipak NH2P-50 4E, 4.6 x 250 mm; Showa Denko K.K., Tokyo, Japan) at a flow rate of 0.6 ml/min at

30ºC. The mobile phase consisted of solvent E (93% acetonitrile and 0.3% acetic acid titrated to pH 7.0 with 1 M aqueous ammonia) and solvent F (20% acetonitrile and 0.3% acetic acid titrated to pH 7.0 with 1 M aqueous ammonia). The column was equilibrated with mixtures of solvent E and solvent F (80:20) that were linearly increased to 49% in 240 min and then to 90% in 7 min. NP-HPLC analysis was performed using the Prominence HPLC system equipped with a fluorescence detector (excitation and emission wavelengths were 310 and 380 nm, respectively; Shimadzu, Kyoto, Japan). Each detected PA-*N*-glycan was further analyzed by RP-HPLC. RP-HPLC was performed on a Develosil C30-UG-5 column (4.6 x 150 mm; Nomura Chemical, Seto, Japan) at a flow rate of 0.5 ml/min at 30ºC. Solvent G consisted of 5 mM ammonium acetate buffer (pH 4.0), and solvent H consisted of solvent G containing 10% acetonitrile. The column was equilibrated with mixtures of solvent G and solvent H (initial ratio 75:25) that were increased linearly to 47% in 55 min and then to 70% in 5 min. PA-sugar chains were detected at excitation and emission wavelengths of 320 and 400 nm, respectively. *N*-glycan structures were identified by calculating the Mannose-Unit value from NP-HPLC, and the Glucose-Unit value from RP-HPLC, as described previously[7,8], or by comparison with known standards and sequential exoglycosidase digestion.

Identification of the anionic *N*-glycans in mouse PNS myelin

The anionic fractions of the major peaks 5-9 in Fig. 2A were individually collected and further analyzed. The *N*-glycans from peaks 5 and 6 were desialylated by neuraminidase. After neuraminidase treatment, the neutral *N*-glycans were collected using DEAE HPLC (Supplementary Figs. S2A and S2B) and analyzed by NP-HPLC (Figs. 2B and 2C). After major peaks 5a, 5b, 5c (from peak 5) and 6' (from peak 6) were collected, the *N*-glycans of these peaks were further analyzed and identified by RP-HPLC using known standards (Supplementary Figs. S3A-S3D). The sialylated *N*-glycan structures from peaks 5 and 6 are shown in Fig. 2F. After desialylation by neuraminidase, the *N*-glycan peaks 7+8 and 9 shifted to the elution position of mono-sulfated *N*-glycans on DEAE HPLC (Supplementary Figs. S2C and S2D). These mono-sulfated *N*-glycans were collected and desulfated by mild methanolysis. After desulfation, the neutral *N*-glycans were collected using DEAE HPLC (Supplementary Figs. S2C and S2D) and analyzed by NP-HPLC (Figs. 2D and 2E). The major peaks (7+8)i, (7+8)ii (from peaks 7+8) and 9' (from peak 9) were collected and further analyzed by RP-HPLC (Supplementary Figs. S3E-S3G). The *N*-glycan elution profiles of peaks (7+8)i, (7+8)ii and 9' coincided with those of the peaks 5a, 5b and 5c, respectively (Figs. 2B, 2D, 2E and

Supplementary Figs. S3A-S3C, S3E-S3G). The sialylated *N*-glycans from peaks 7+8 and 9 were α2,3-sialidase-resistant, but neuraminidase-sensitive. After the *N*-glycans from peaks 7+8 were treated with neuraminidase, the *N*-glycan elution profiles coincided with GP3 and desialylated GP4 profiles on RP-HPLC. Taken together, the presumed *N*-glycan structure from peaks 7+8 and 9 are shown in Fig. 2F. There are two possibilities for the *N*-glycan structure from peak 9.

Identification of the main neutral *N*-glycan in mouse PNS myelin

The neutral fraction in Fig. 2A was collected using DEAE HPLC and analyzed by NP-HPLC (Supplementary Fig. S4A). After the fraction of the main peak N1 was collected, the *N*-glycans from peak N1 were further separated by RP-HPLC (Supplementary Fig. S4B). The main peak N2 was identified using a known standard. This neutral *N*-glycan structure is shown in Supplementary Fig. S4C.

Data quantification and analysis of PA-*N*-glycans

PA-*N*-glycans were quantified and analyzed as described previously[5,6]. HPLC chromatogram data were analyzed using LC station software (Shimadzu) and Empower2 software (Waters, Milford, MA).

RT-PCR

Total RNA was extracted from sciatic nerves and the medulla oblongata of 12-week-old young adult mice with Sepasol-RNA I Super (Nacalai Tesque) according to the manufacturer's instructions. cDNA was synthesized from 1 μg of total RNA using ReverTra Ace reverse transcriptase (Toyobo, Osaka, Japan) with random 6-mer primers. The retrotranscription reaction was subjected to PCR amplification using the following primers and KAPA Taq EXtra (Nippon Genetics, Tokyo, Japan) in a thermal cycler. The primers used were as follows (from 5' to 3'): GlcNAc6ST-1 (Chst2: forward, CCTGAGGTGTTCTTCCTCTATGA; reverse, CGTACTAGGTGGATGACCTTGAG), GlcNAc6ST-2 (Chst4: forward, GTGTGACATGAGCGTCTTTGAT; reverse, GCAGATGATCTTCATGGCATAAT), GlcNAc6ST-3 (Chst5: forward, GAGGAGGTGTGTAAGCCTCTGT; reverse, GAAGGCATAGAGTTCACGGATTA), GlcNAc6ST-4 (Chst7: forward, TGCTGCGTGACCCAGGCCTCAA; reverse, CATATTGAATGCGAAGGCATC), and β-actin (forward, TGACAGGATGCAGAAGGAGA; reverse, GCTGGAAGGTGGACAGTGAG). Aliquots of each reaction product were separated by electrophoresis

through a 2% agarose gel containing ethidium bromide.

Immunofluorescence studies

Immunostaining was performed as described previously[9]. Mice were anesthetized and fixed by transcardial perfusion with 4% paraformaldehyde (PFA) in 0.1 M PB (pH 7.4). The sciatic nerves were then postfixed in the same fixative. Frozen sections (10 μm) were prepared. The teased nerve fibers and frozen sections were treated with methanol for 20 min at 4°C. After three washes with 0.01 M phosphate-buffered saline (PBS), sections were incubated with 5% BSA in 0.01 M PBS (pH 7.4) containing 0.1% Triton X- 100 (PBS-T) for 1 hr at room temperature to block nonspecific binding. The slides were then incubated overnight at 4ºC with primary antibodies (anti-Caspr polyclonal antibody, kindly provided by Dr. E. Peles, The Weizmann Institute of Science, Rehovot, Israel) diluted to appropriate concentrations in PBS-T. After three 5-min washes in PBS, the slides were incubated for 1 hr at room temperature with secondary antibodies (Alexa 488-labeled goat anti-rabbit IgG, Molecular Probes, Thermo Fisher Scientific, Waltham, MA) followed by three washes with PBS. For fluorescence-labeled secondary antibodies, images were captured with either a fluorescence microscope Axio Imager (Zeiss, Oberkochen, Germany) or a laser scanning microscope LSM5 PASCAL (Zeiss). Digitized images were transferred to a laboratory computer for later analysis using AxioVision (Zeiss).

Morphological studies

Mice were anesthetized and perfused intracardially through the left ventricle with a fixative solution consisting of 4% PFA and 1.25% glutaraldehyde in 0.1 M PB, pH 7.4. Dissected sciatic nerves were transferred to a fixative containing 2.5% glutaraldehyde and 2.0% PFA in 0.1 M sodium cacodylate trihydrate buffer (cacodylate buffer), pH 7.4. After osmification in a 1% osmium tetroxide solution, the specimens were dehydrated through a graded alcohol series and embedded in Epon 812 (TAAB Laboratories, Aldermaston, UK). Semi-thin cross sections (0.7 μm) were stained with 0.5% Toluidine blue in 10 mM PBS (pH 7.4) and analyzed using the Axio Imager (Zeiss). The g-ratio was measured by dividing the diameter of an axon (without myelin) by that of the total fiber diameter (axon + myelin sheath) using ImageJ. Diameters were normalized by their perimeters.

SBF-SEM imaging and analyses

The imaging and 3D ultrastructural analyses were performed as described previously[10,11]. Briefly, sciatic nerves of 6 wild-type and 6 GlcNAc6ST-1-KO mice were removed after transcardial perfusion using PBS and 0.1M PB (pH 7.4) containing 4% PFA and 0.5% glutaraldehyde. Tissues were immersed in the same fixative overnight. The samples were post-fixed with reduced osmium, stained *en bloc* with thiocarbohydrazide, osmium and lead, and embedded in conductive resin. Following trimming, samples were imaged with Sigma VP or Merlin (Zeiss) equipped with 3View2XP (Gatan, Pleasanton, CA). The serial images acquired were handled and processed for segmentation and 3D reconstruction with Fiji/ImageJ (http://fiji.sc/Fiji) and Amira (FEI Visualization Science Group, Hillsboro, OR).

Supplementary References

1. Sedzik, J., Kotake, Y. & Uyemura, K. Purification of P0 myelin glycoprotein by a Cu^{2+}-immobilized metal affinity chromatography. *Neurochem. Res.* **24**, 723-732 (1999).
2. Sedzik, J., Uyemura, K. & Tsukihara, T. Towards crystallization of hydrophobic myelin glycoproteins: P0 and PASII/PMP22. *Protein Expr. Purif.* **26**, 368-377 (2002).
3. Yamaguchi, Y., Miyagi, Y. & Baba, H. Two-dimensional electrophoresis with cationic detergents, a powerful tool for the proteomic analysis of myelin proteins. Part 1: technical aspects of electrophoresis. *J. Neurosci. Res.* **86**, 755-765 (2008).
4. Yamaguchi, Y., Miyagi, Y. & Baba, H. Two-dimensional electrophoresis with cationic detergents: a powerful tool for the proteomic analysis of myelin proteins. Part 2: analytical aspects. *J. Neurosci. Res.* **86**, 766-775 (2008).
5. Yoshimura, T. *et al.* Detection of *N*-glycans on small amounts of glycoproteins in tissue samples and sodium dodecyl sulfate-polyacrylamide gels. *Anal. Biochem.* **423**, 253-260 (2012).
6. Torii, T. *et al.* Determination of major sialylated *N*-glycans and identification of branched sialylated *N*-glycans that dynamically change their content during development in the mouse cerebral cortex. *Glycoconj. J.* **31**, 671-683 (2014).
7. Hase, S. High-performance liquid chromatography of pyridylaminated saccharides. *Methods Enzymol.* **230**, 225-237 (1994).
8. Otake, Y. *et al.* Isolation and characterization of an *N*-linked oligosaccharide that is significantly increased in sera from patients with non-small cell lung cancer. *J. Biochem.* **129**, 537-542 (2001).
9. Hayashi, A. *et al.* Localization of annexin II in the paranodal regions and Schmidt-Lanterman incisures in the peripheral nervous system. *Glia* **55**, 1044-1052 (2007).
10. Nguyen, H. B. *et al.* Conductive resins improve charging and resolution of acquired images in electron microscopic volume imaging. *Sci. Rep.* **6**, 23721 (2016).
11. Thai, T. Q. *et al.* Rapid specimen preparation to improve the throughput of electron microscopic volume imaging for three-dimensional analyses of subcellular ultrastructures with serial block-face scanning electron microscopy. *Med. Mol. Morphol.* **49**, 154-162 (2016).

Supplementary figure legends

Supplementary Figure S1. Anionic *N*-glycans are abundant in the PNS, but not CNS.

(A) PA-*N*-glycans from bovine PNS myelin were separated using a DEAE column. The anionic *N*-glycans of peaks 1-4 are shown in Fig. 1H. (B) PA-*N*-glycans from mouse CNS myelin were analyzed by DEAE HPLC. (C) PA-*N*-glycans from adult rat sciatic nerves were separated using a DEAE column. The asterisk indicates the peak derived from contaminants. Arrowheads indicate the elution positions of mono-, di-, tri-, and tetra-sialyl standard PA-oligosaccharides.

Supplementary Figure S2. Peaks are shifted on DEAE HPLC after desialylation.

(A-D) After the PA-*N*-glycans from peaks 5, 6, 7+8 and 9 in Fig. 2A were desialylated by neuraminidase, peaks 5 (A) and 6 (B) were shifted to the elution position of neutral *N*-glycans, and peaks 7+8 (C) and 9 (D) were shifted to that of mono-sulfated *N*-glycans by DEAE HPLC (blue). The neutral *N*-glycans of the desialylated peaks 5 (A) and 6 (B) were collected and further analyzed by NP-HPLC (Figs. 2B and 2C). The mono-sulfated *N*-glycans of the desialylated peaks 7+8 (C) and 9 (D) were collected and desulfated by mild methanolysis. After desulfation, the peaks were shifted to the elution position of neutral *N*-glycans (red). These neutral *N*-glycans were collected and further analyzed by NP-HPLC (Figs. 2D and 2E). Arrowheads indicate the elution positions of mono-, di-, tri-, and tetra-sialyl standard PA-oligosaccharides.

Supplementary Figure S3. Identification of *N*-glycans by RP-HPLC.

(A-G) The PA-*N*-glycans from peaks 5a (A), 5b (B), 5c (C), 6' (D), (7+8)i (E), (7+8)ii (F) and 9' (G) in Figs. 2B-2E were analyzed by RP-HPLC. There was one main peak in each *N*-glycan elution profile. The *N*-glycan elution profiles of peaks 6'', (7+8)i', (7+8)ii' and 9'' coincided with those of the peaks 5c', 5a', 5b' and 5c', respectively. The *N*-glycans were identified using known standards (structures shown in Fig. 2F).

Supplementary Figure S4. Identification of the most abundant neutral *N*-glycan in PNS myelin.

(A) The neutral PA-*N*-glycans in Fig. 2A were analyzed by NP-HPLC. The main peak N1 was collected for further analysis. (B) The PA-*N*-glycans from peak N1 were further separated by RP-HPLC. The main peak N2 was identified using a known standard. (C) The structure of the most abundant neutral *N*-glycan in mouse

PNS myelin is shown as a PA-form.

Supplementary Figure S5. GlcNAc6ST-1 mRNA is expressed in the CNS.

The mRNA expression levels of GlcNAc6ST-1 and β-actin in the medulla oblongata (CNS) and sciatic nerves (PNS) were analyzed by RT-PCR.

Supplementary Figure S6. GlcNAc6ST-1-null mice fail to sulfate *N*-glycans in PNS myelin.

PA-*N*-glycans from PNS myelin of adult GlcNAc6ST-1-KO mice (red) were separated using a DEAE column. The elution positions of peaks 5-8 coincided with those from PNS myelin of WT mice (black). The asterisk indicates the peak derived from contaminants. Arrowheads indicate the elution positions of mono-, di-, tri-, and tetra-sialyl standard PA-oligosaccharides.

Supplementary Figure S7. *N*-glycans on P_0 protein are not sulfated in CNS myelin.

(A-C) PA-*N*-glycans in CNS myelin from WT (A), P_0-CNS (B) and PLP-null (C) mice were analyzed by DEAE HPLC. In P_0-CNS mice, P_0 protein is highly expressed in CNS myelin instead of PLP. The anionic peaks are mainly derived from sialylated *N*-glycans. The asterisk indicates the peak derived from contaminants. Arrowheads indicate the elution positions of mono-, di-, tri-, and tetra-sialyl standard PA-oligosaccharides.

Supplementary Figure S1

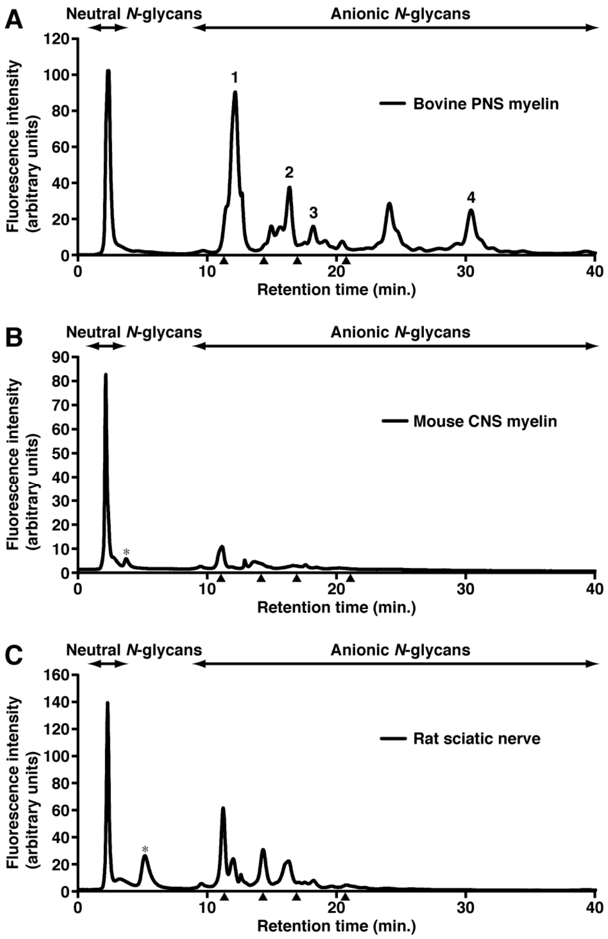

Supplementary Figure S2

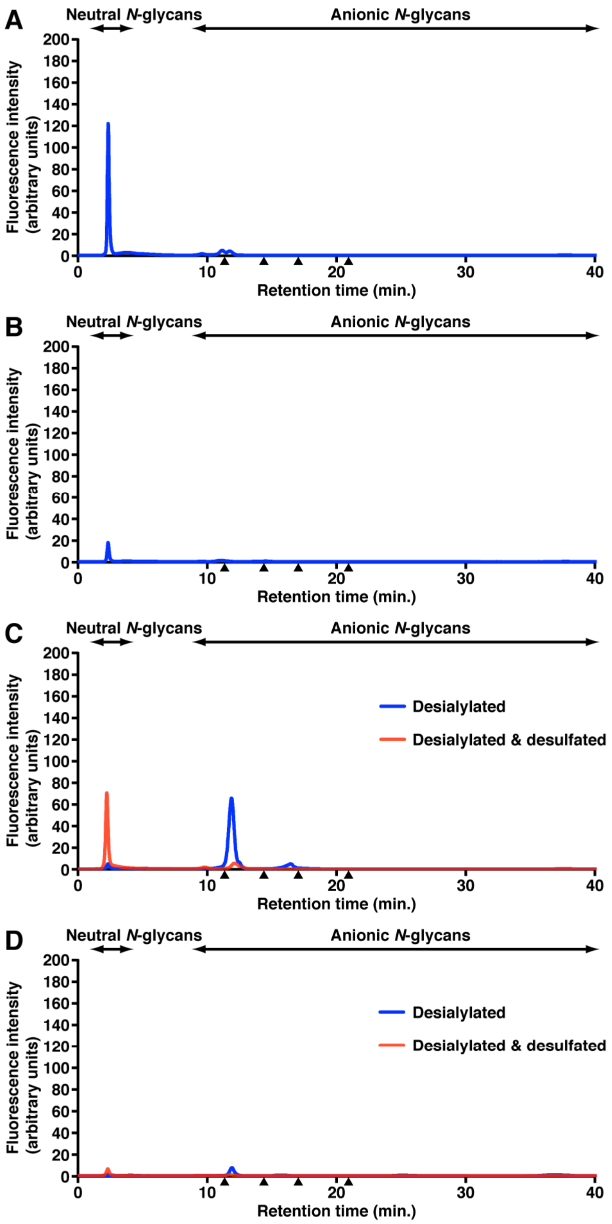

Supplementary Figure S3

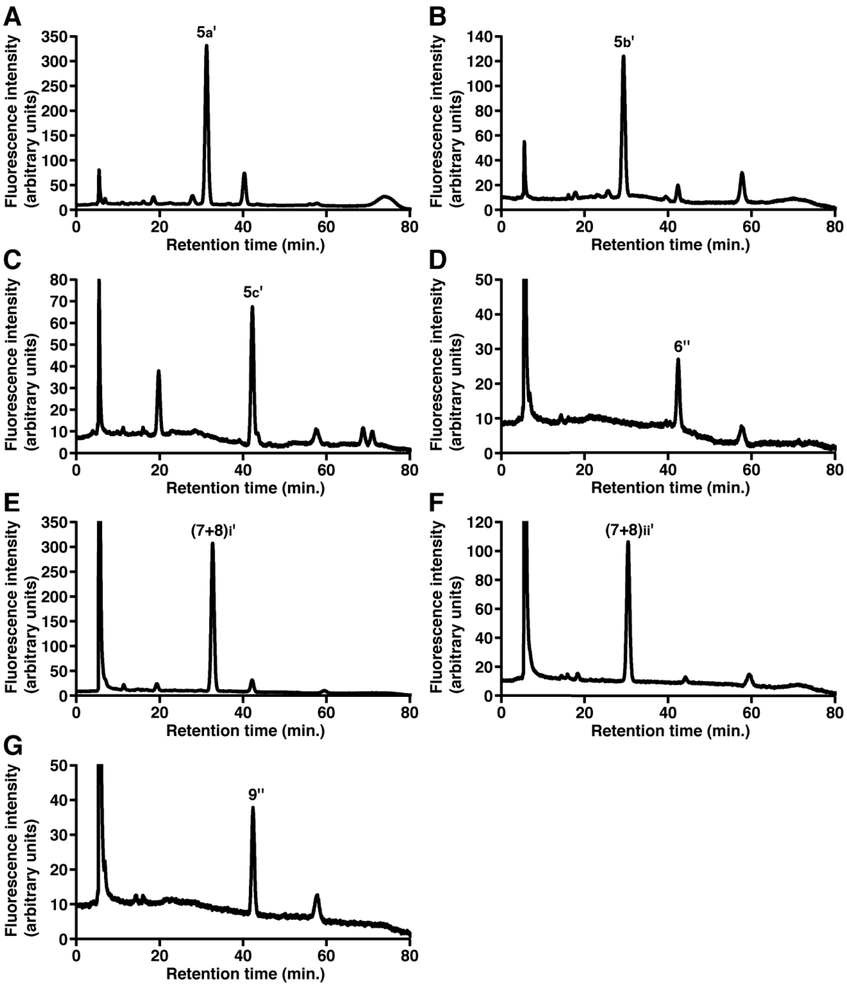

Supplementary Figure S4

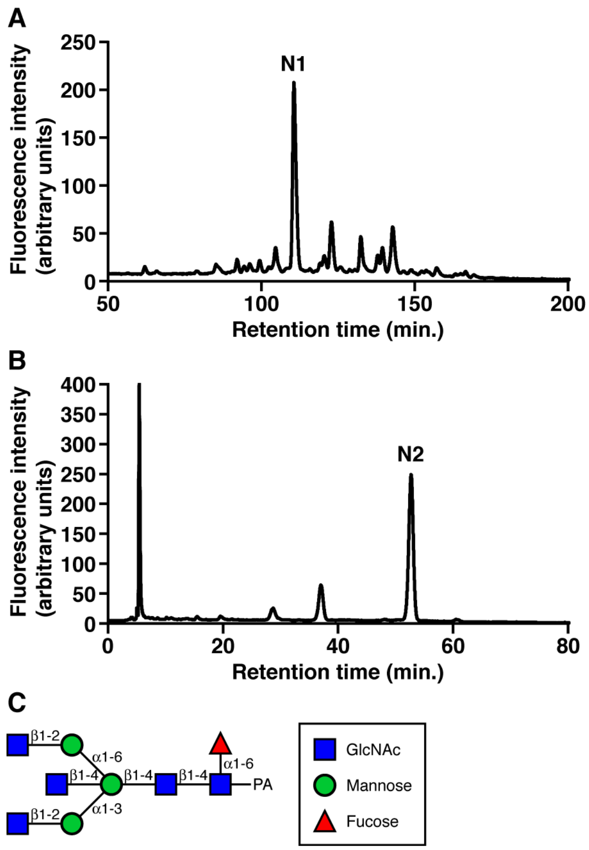

Supplementary Figure S5

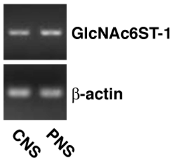

Supplementary Figure S6

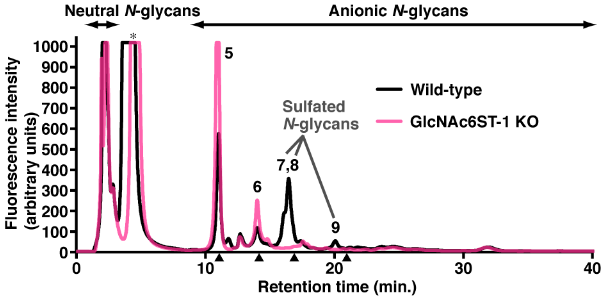

Supplementary Figure S7

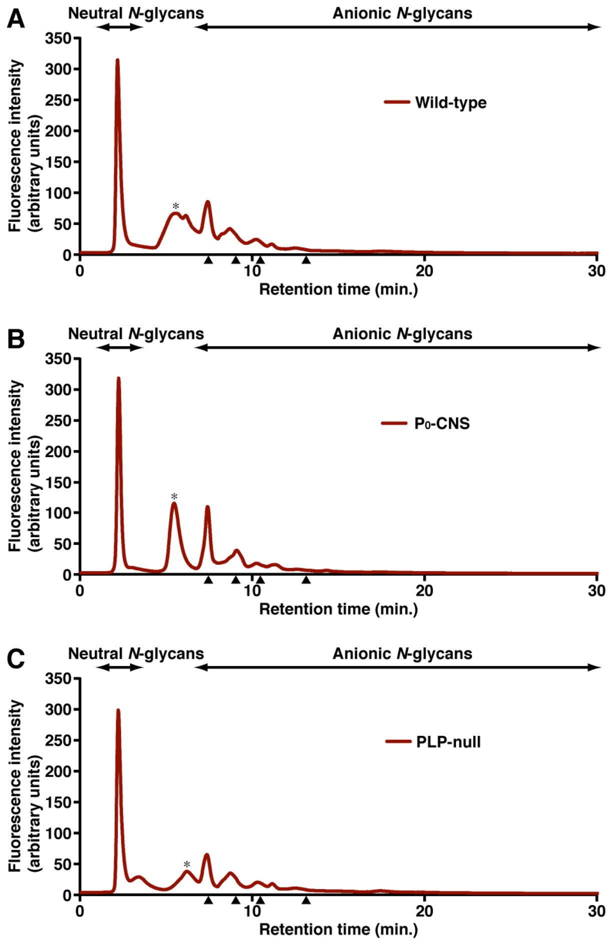

Supplement: Supplementary Information [file srep42257-s1.pdf]
